# Genetic Variation in the *EGFR* Gene and the Risk of Glioma in a Chinese Han Population

**DOI:** 10.1371/journal.pone.0037531

**Published:** 2012-05-25

**Authors:** Wu-Gang Hou, Wen-Bo Ai, Xiao-Guang Bai, Hai-Long Dong, Zhen Li, Yuan-Qiang Zhang, Li-Ze Xiong

**Affiliations:** 1 Department of Anesthesiology, Xijing Hospital, The Fourth Military Medical University, Xi'an, China; 2 Department of Health Service, School of Preventive Medicine, The Fourth Military Medical University, Xi'an, China; 3 Department of Histology and Embryology, The Fourth Military Medical University, Xi'an, China; Dartmouth College, United States of America

## Abstract

Previous studies have shown that regulation of the epidermal growth factor gene (*EGFR*) pathway plays a role in glioma progression. Certain genotypes of the *EGFR* gene may be related to increased glioblastoma risk, indicating that germ line *EGFR* polymorphisms may have implications in carcinogenesis. To examine whether and how variants in the *EGFR* gene contribute to glioma susceptibility, we evaluated nine tagging single-nucleotide polymorphisms (tSNPs) of the *EGFR* gene in a case–control study from Xi'an city of China (301 cases, 302 controls). EGFR SNP associations analyses were performed using SPSS 16.0 statistical packages, PLINK software, Haploview software package (version 4.2) and SHEsis software platform. We identified two susceptibility tSNPs in the *EGFR* gene that were potentially associated with an increased risk of glioma (rs730437, *p* = 0.016; OR: 1.32; 95%CI: 1.05–1.66 and rs1468727, *p* = 0.008; OR: 1.31; 95%CI: 1.04–1.65). However, after a strict Bonferroni correction analysis was applied, the significance level of the association between EGFR tSNPs and risk of glioma was attenuated. We observed a protective effect of haplotype “AATT” of the *EGFR* gene, which was associated with a 29% reduction in the risk of developing glioma, while haplotype “CGTC” increased the risk of developing glioma by 36%. Our results, combined with previous studies, suggested an association between the *EGFR* gene and glioma development.

## Introduction

According to the classification by the World Health Organization (WHO), glioma encompasses all tumors that are thought to be of glial cell origin, including Astrocytic tumors [Astrocytoma grades I, II (Astrocytoma), III (Anaplastic astrocytoma), and IV (Glioblastoma or GBM)], Oligodendrogliomas, Ependymomas, and Mixed gliomas (Central Brain Tumor Registry of the United States). Gliomas are common tumors and account for almost 80% of primary malignant brain tumors, usually resulting in poor survival compared to other types of brain tumors.

Current evidence suggests that inherited risks play a role in glioma susceptibility, as with other cancers. A majority of the inherited risk is due to the co-inheritance of multiple low-risk variants, some of which are commonly seen gene variants and hence can be identified through association studies [1]. The epidemiology of glioma has focused on identifying factors that can be modified to prevent this disease [2]–[4]. Recent research has focused on identifying germ line polymorphisms associated with the risk of glioma and defining molecular markers to classify glial tumors in more homogenous groups [2]–[4].

The epidermal growth factor receptor (*EGFR*) regulates important cellular processes and is implicated in human tumors. Several previous studies have assessed single nucleotide polymorphisms (SNPs) in the *EGFR* gene for the association with the risk of cancers, such as lung cancer [5], [6], breast cancer [7], prostate cancer [8], and esophageal cancer [9]. Somatic alterations of the *EGFR* gene are common in glioma and influence several mechanisms of malignant transformation [10]. Previous studies have shown that regulation of the *EGFR* pathway plays an important role in glioma progression [11], and certain *EGFR* genotypes may be related to glioblastoma risk, indicating that germline *EGFR* polymorphisms may have important implications in carcinogenesis of glioma [12].

In addition, it is possible that haplotypes and locus–locus interactions within the *EGFR* gene may be correlated with the development of glioma. To investigate potential relationships between *EGFR* SNP polymorphisms, haplotypes, locus–locus interactions, and their role in the etiology of gliomas, we performed a comprehensive association analysis in a case–control study in the Han Chinese population. Our study indicated important evidence for the association between *EGFR* gene polymorphisms and the risk of glioma.

## Results

A total of 301 cases (157 male, 144 female; median age at diagnosis 41.5 yrs) and 302 controls (155 male, 147 female; median age 42.3 yrs) were included in the current study. Basic characteristics of the cases and controls were listed in Table 1 including gender, age, and pathology. As listed in Table 2, a multiplexed SNP MassEXTEND assay was designed with the Sequenom MassARRAY Assay Design 3.0 Software. Nine SNPs in the *EGFR* gene in glioma patients and the control group were genotyped (raw genotype data are listed in Table S1 and Table S2). The average tSNPs call rate was 98.5% in cases and controls. All of the tested tSNPs are in Hardy–Weinberg equilibrium (HWE) in the control population of this study (Table 3). We compared the differences in frequency distributions of alleles between cases and controls by χ^2^ test and found two significant tSNPs in the *EGFR* gene at a 5% level (rs1468727, *p* = 0.008, odds ratio [OR]: 1.31, 95% confidence interval [CI]: 1.04–1.65 and rs730437, *p* = 0.016, OR: 1.32, 95%CI: 1.05–1.66). After a strict Bonferroni correction analysis was applied, we found no association between *EGFR* tSNPs and risk of glioma (Table 3). We further analyzed the allele frequency differentiation of rs730437 and rs1468727 between diverse groups of cases with varying aggressive grades and found no association between tumor aggressiveness and presence of the risk allele (Table S3).

**Table 1:** Basic characteristics of case and control patients.

	Cases (n = 301)		Controls (n = 302)		*P* value from χ^2^
	No.	%	No.	%	
Sex					0.837
Male	157	52.2	155	51.3	
Female	144	47.8	147	48.7	
Age					0.063
> = 50	117	38.9	140	46.4	
<50	184	61.1	162	53.6	
Median age	41.5		42.3		
Histologic type					
Astrocytoma	173	57.5			
Ependymoma	20	6.6			
Glioblastoma	42	14.0			
Oligodendroglioma	9	3.0			
others	57	18.9			

**Table 2:** PCR primers.

SNP_ID	1st-PCR primer sequences	2nd-PCR primer sequences	UEP sequences
rs730437	ACGTTGGATGAGGGAACCAGGCGCAGGTCA	ACGTTGGATGAGTGTGAGCTTGCGTCTCAG	CAGTGCTGGCCTGAG
rs845552	ACGTTGGATGTCCAACTGTGCGCTCTGCCT	ACGTTGGATGGCAAGCATGCTTGGTATTCC	TGGTATTCCACAACAATCT
rs1468727	ACGTTGGATGCCACAGCTTGGATCCAGAAA	ACGTTGGATGGCCTATCAGCTAAAGGATTC	ACTTGGTCCTCTTATCCT
rs3752651	ACGTTGGATGACTTCCAGGAAAAGAGATTC	ACGTTGGATGGCACAATAGGAAATAAGCAAG	ATATGAAATAAGCAAGTATTATTGCC
rs4947492	ACGTTGGATGTCGTGGTTCCTGTTCATCTG	ACGTTGGATGACCAGGAAGTGGAGATAGTC	AGTGGAGATAGTCACATATTAGCC
rs9642393	ACGTTGGATGATCTGATAGACCCACTGGGC	ACGTTGGATGAACGGGACACACGACTGAAC	AGGAACAGCGTTCCCAT
rs11506105	ACGTTGGATGGAGCAAAGGTTCCCTGTGAG	ACGTTGGATGGAAAAAGTCTGCAAGTGCTC	TCCCCAGTCTGCAAGTGCTCTGCGAC
rs12718945	ACGTTGGATGTAGTTTTCTCAATCCCATG	ACGTTGGATGTGTTTCAAGTTGGGAGAAGG	GGAGAAGGAGATTATTTAATACTAAAA
rs17172432	ACGTTGGATGTTTCCTCATGGGACACATGG	ACGTTGGATGGGAATTTACTATCAAATCTC	CAATTTACTATCAAATCTCAGTTGTTA

*UEP: Unextended mini-sequencing primer.*

**Table 3:** Examined tSNPs examined in the *EGFR* gene.

SNP_ID	Location	Position (Genome build 36.3)	HWE *p* value	*p* value from χ^2^	*p* value adj.*	OR (95%CI)
rs11506105	7p11.2	55187671(boundary)	0.909	0.053	0.477	1.23(0.97–1.56)
rs12718945	7p11.2	55160457(Intron 1)	0.904	0.777	1	1.04(0.82–1.32)
rs1468727	7p11.2	55197599(Intron 13)	0.757	0.008	0.072	1.31(1.04–1.65)
rs17172432	7p11.2	55108811 (Intron 1)	0.926	0.563	1	0.88(0.61–1.28)
rs3752651	7p11.2	55197037(Intron 13)	0.925	0.232	1	1.11(0.73–1.69)
rs4947492	7p11.2	55155486(Intron 1)	0.882	0.723	1	1.04(0.82–1.32)
rs730437	7p11.2	55182512(Intron 4)	0.960	0.016	0.144	1.32(1.05–1.66)
rs845552	7p11.2	55213001(Intron 19)	0.643	0.105	0.945	1.24(0.98–1.56)
rs9642393	7p11.2	55213141(Intron 19)	0.979	0.115	1	1.2(0.95–1.51)

Note:

* *p* value was adjusted by Bonferroni corrections.

Association results between *EGFR* tSNP genotypes and the risk of glioma were listed in Table 4. We identified two significant SNP genotypes associated with the risk of glioma, one was genotype “CC” of rs1468727 (OR, 1.78; 95% CI, 1.11–2.84; *p* = 0.016) and the other was genotype “CC” of rs730437 (OR, 1.74; 95% CI, 1.07–2.83; *p* = 0.024).

**Table 4:** Association between *EGFR* tSNP genotypes and the risk of glioma.

SNP_ID	Genotype	No. (frequency)		OR (95% CI) *p* value	
		Case	Control		
rs11506105	GG	50(16.9)	37(12.5)	1.56(0.94–2.56)	0.081
	AG	140(47.3)	137(46.3)	1.18(0.83–1.67)	0.365
	AA	106(35.8)	122(41.2)	1(referent)	-
rs12718945	TT	36(12.1)	35(11.7)	1.07(0.63–1.81)	0.801
	GT	138(46.3)	135(45.2)	1.06(0.76–1.5)	0.725
	GG	124(41.6)	129(43.1)	1(referent)	-
rs1468727	CC	77(25.8)	50(17.2)	1.78(1.11–2.84)	0.016
	TC	143(48)	150(51.7)	1.1(0.75–1.61)	0.623
	TT	78(26.2)	90(31)	1(referent)	-
rs17172432	CC	2(0.7)	4(1.3)	0.48(0.09–2.67)	0.659
	CT	54(17.9)	56(18.9)	0.93(0.62–1.41)	0.742
	TT	245(81.4)	237(79.8)	1(referent)	-
rs3752651	CC	2(0.7)	1(0.3)	2.02(0.18–22.37)	0.997
	CT	46(15.3)	43(14.4)	1.08(0.69–1.69)	0.743
	TT	252(84)	254(85.2)	1(referent)	-
rs4947492	GG	37(12.3)	36(12)	1.07(0.64–1.8)	0.800
	GA	141(46.8)	137(45.5)	1.07(0.76–1.51)	0.694
	AA	123(40.9)	128(42.5)	1(referent)	-
rs730437	CC	56(18.6)	40(13.3)	1.74(1.07–2.83)	0.024
	CA	147(48.8)	139(46.2)	1.32(0.93–1.87)	0.126
	AA	98(32.6)	122(40.5)	1(referent)	-
rs845552	AA	57(19.1)	43(14.8)	1.5(0.93–2.4)	0.094
	GA	132(44.3)	125(43)	1.19(0.84–1.7)	0.333
	GG	109(36.6)	123(42.3)	1(referent)	-
rs9642393	TT	57(19.5)	43(14.5)	1.48(0.92–2.39)	0.106
	CT	135(46.1)	140(47.3)	1.08(0.75–1.54)	0.677
	CC	101(34.5)	113(38.2)	1(referent)	-

OR: odd ratio; CI: confidence interval.

We assumed that the minor allele of each tSNP was a risk allele compared to the wild type allele. Minor allele frequency (MAF) in cases and controls were listed in Table 5. Further model association analyses were performed by logistic tests. The rs730437 was observed to be associated with glioma risk by both recessive model analyses (OR, 1.68; 95% CI, 1.04–2.69; *p* = 0.032) and additive model analyses (OR, 1.35; 95% CI, 1.05–1. 72; *p* = 0.019). We also observed another susceptibility SNP, rs1468727, by recessive model analyses (OR, 1.88; 95% CI, 1.22–2.89; *p* = 0.004) and additive model analyses (OR, 1.37; 95% CI, 1. 07–1.76; *p* = 0.012).

**Table 5:** Association between *EGFR* tSNPs and the risk of glioma based on logistic tests and their heterozygote and homozygote odds ratios, per allele odds ratios and confidence intervals.

SNP No.	Minor Allele	MAF Case	MAF Control	Dominant Model				Recessive Model				Additive Model			
				OR	95% CI		*p*	OR	95% CI		*p*	OR	95% CI		*p*
rs11506105	G	0.41	0.36	1.25	0.88	1.78	0.218	1.58	0.97	2.58	0.069	1.26	0.98	1.62	0.071
rs12718945	T	0.35	0.34	1.03	0.73	1.45	0.881	1.08	0.64	1.83	0.781	1.03	0.80	1.33	0.807
rs1468727	C	0.50	0.43	1.29	0.88	1.89	0.190	1.88	1.22	2.89	0.004	1.37	1.07	1.76	0.012
rs17172432	C	0.10	0.11	0.92	0.60	1.42	0.702	0.61	0.11	3.38	0.571	0.91	0.61	1.35	0.624
rs3752651	C	0.08	0.08	1.09	0.67	1.75	0.739	4.52	0.38	53.81	0.233	1.14	0.72	1.79	0.579
rs4947492	G	0.36	0.35	1.02	0.72	1.44	0.917	1.11	0.66	1.87	0.701	1.04	0.80	1.33	0.793
rs730437	C	0.43	0.36	1.38	0.97	1.97	0.077	1.68	1.04	2.69	0.032	1.35	1.05	1.72	0.019
rs845552	A	0.41	0.36	1.34	0.94	1.91	0.105	1.34	0.84	2.12	0.221	1.24	0.97	1.58	0.081
rs9642393	T	0.42	0.38	1.25	0.87	1.79	0.225	1.38	0.87	2.20	0.169	1.22	0.95	1.56	0.119

MAF: minor allele frequency; OR: odd ratio; CI: confidence interval.

**Figure 1:**
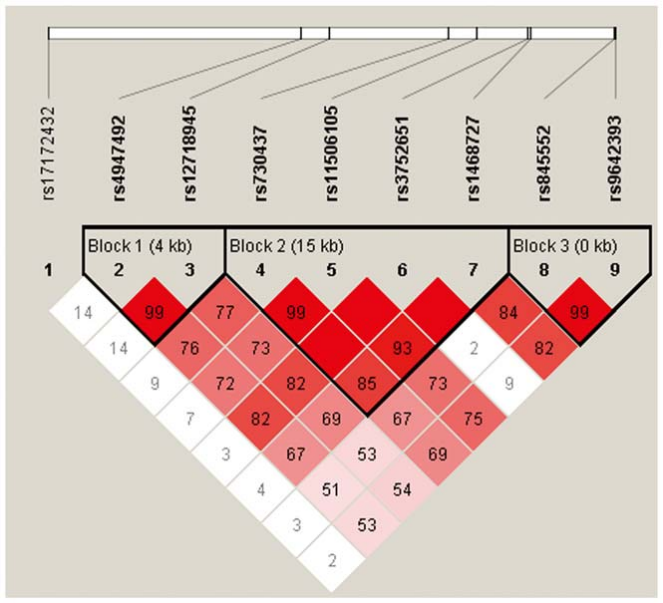
Haplotype block map for all the tSNPs of the *EGFR* gene. Block 1 includes rs4947492 and rs12718945; Block 2 includes rs730437, rs11506105, rs3752651 and rs1468727; and Block 3 includes rs845552 and rs9642393. The LD between two SNPs is standardized D′ (red schemes).

Three blocks were detected in studied *EGFR* SNPs by haplotype analyses (Figure 1). The global result for Block 1 (rs4947492 and rs12718945) was: total case = 594, total control = 596, global χ^2^ = 0.106 while df = 1, Fisher's *p* value = 0.744, and Pearson's *p* value = 0.744. The global result for Block 2 (rs730437, rs11506105, rs3752651, and rs1468727) was: total case = 582, total control = 559, global χ^2^ = 6.584 while df = 2, Fisher's *p* value = 0.037, and Pearson's *p* value = 0.037. The global result for Block 3 (rs845552 and rs9642393) was: total case = 578, total control = 572, global χ^2^ = 2.79 while df = 1, Fisher's *p* value = 0.095, and Pearson's *p* value = 0.095. The global result was: total case = 545, total control = 517, global χ^2^ = 18.814 while df = 6, Fisher's *p* value = 0.005, and Pearson's *p* value = 0.005 (frequency <0.03 in both the control and case was dropped.).

The results of the association between the *EGFR* haplotype and the risk of glioma were listed in Table 6. Haplotype “CGTC” in Block 2 was found to be associated with the risk of glioma (OR, 1.321; 95% CI, 1.033–1.688; Fisher's *p* = 0.026; Pearson's *p* = 0.026). In Block 2, we also found a protective haplotype “AATT” associated with the risk of glioma (OR, 0.732; 95% CI, 0.576–0.929; Fisher's *p* = 0.01; Pearson's *p* = 0.01). Global haplotype association analyses showed that haplotype “TGTAATTGC” was associated with an increased risk of glioma at a 1% level (OR, 0.286; 95% CI, 0.135–0.609; Fisher's *p* = 0.001; Pearson's *p* = 0.001).

**Table 6:** *EGFR* haplotype frequencies and the association with the risk of glioma in case and control patients.

Block	Haplotype	freq(case)	freq(control)	χ^2^	Fisher's *p*	Pearson's *p*	OR	[95%CI]
1	A G	0.645	0.653	0.106	0.744	0.744	0.961	[0.757,1.221]
	G T	0.352	0.342	0.106	0.744	0.744	1.041	[0.819,1.322]
2	A A C C	0.084	0.073	0.514	0.474	0.474	1.171	[0.76,1.804]
	A A T T	0.471	0.546	6.571	0.01	0.01	0.732	[0.576,0.929]
	C G T C	0.394	0.333	4.945	0.026	0.026	1.321	[1.033,1.688]
3	A T	0.412	0.362	2.79	0.095	0.095	1.226	[0.965,1.556]
	G C	0.578	0.622	2.79	0.095	0.095	0.816	[0.643,1.036]
Total	C A G A A T T G C	0.04	0.048	0.515	0.473	0.473	0.808	[0.452,1.447]
	T A G A A C C G C	0.046	0.028	2.359	0.125	0.125	1.661	[0.864,3.193]
	T A G A A T T G C	0.395	0.421	1.669	0.196	0.196	0.843	[0.65,1.093]
	T A G C G T C A T	0.086	0.063	1.831	0.176	0.176	1.374	[0.866,2.18]
	T G T A A T T G C	0.016	0.053	11.841	0.001	0.001	0.286	[0.135,0.609]
	T G T C G T C A T	0.229	0.188	2.239	0.135	0.135	1.258	[0.931,1.7]
	T G T C G T C G C	0.036	0.027	0.530	0.467	0.467	1.29	[0.649,2.564]

OR: odd ratio; CI: confidence interval.

## Discussion

In this case–control study in a Han Chinese population, we identified for the first time rs1468727 and rs730437 in the *EGFR* gene associated with an increased risk of glioma. A protective effect was also observed for the haplotype “AATT” of the *EGFR* gene that was associated with a 29% reduction in the risk of developing glioma. Additionally, we also observed a strong effect of the “CGTC” haplotype, which increased the risk of developing glioma by 36%.

Our study adopted a genotype and haplotype based approach. To the best of our knowledge, this study was the first haplotype-based study that described the association between tSNPs in the *EGFR* gene and glioma risk in a Chinese population. Previous studies focused only on one or two variants in the *EGFR* gene, which might not sufficiently capture the effect of susceptibility loci in Chinese glioma patients. A haplotype-based association approach is an increasingly accepted approach for genetic association studies [13]. Using this approach, we provided strong support that *EFGR* gene variations contributed to the susceptibility to glioma.

It is important to note two SNPs (rs1468727 and rs730437) and their relationship with glioma risk in this study. We found that genotype “CC” of rs1468727 in intron 13 of the *EGFR* gene was associated with the risk of glioma in Chinese patients. Interestingly, genotype “TT” of rs1468727 was found to be associated with a decreased risk of glioma in a previous study in a European population (OR, 0.61; 95% CI, 0.40–0.93; *p* = 0.017) [12]. These results supported our findings that rs1468727 was a susceptibility loci and the genotype “CC” of this locus was a risk genotype for glioma. Another SNP, rs730437, located in intron 4 of the *EGFR* gene was identified in both studies. In our study, the genotype “CC” of rs730437 was identified as the risk genotype with frequencies of 0.43 in glioma patients and 0.36 in controls. However, in the European population, the risk genotype was “AA” (OR, 1.32; 95% CI, 1.03–1.68; *p* = 0.032), with frequencies of 0.27 in glioma patients and 0.23 in controls [12]. Together, these findings indicate that ethnic differences among the *EGFR* gene variants may affect the development of glioma in diverse populations. Furthermore, tSNPs rs1468727 and rs730437 may have a tight linkage with other functional SNPs. Therefore, the exact location and biological functions of the real causal SNPs in the *EGFR* gene is of great interest and warrants further investigation.

Haplotype analysis suggested that glioma risk was substantially elevated among individuals with specific haplotypes. Block 2 included four SNPs, one in intron 4, one in the intron/exon boundary, and two others in intron 13. “AATT” was a protective haplotype (OR, 0.732), while “CGTC” was a risk haplotype (OR, 1.321). Global haplotype association analyses showed that haplotype “TGTAATTGC” was associated with the risk of glioma at a 1% level (OR, 0.286; *p* = 0.001), indicating the complexity of this gene in the development of glioma.

Some limitations were inherent in this case–control study and must be noted. The sample size (301 glioma patients and 302 control subjects) was not relatively large among glioma association studies published to date [2]–[4]. Glioma patients were not sub-grouped by age or gender, and gender-specific significant variants were not tested. We selected tSNPs with MAF higher than 5% in HapMap Asian populations to affirm the statistical power was large enough for analyzing data. We performed Bonferroni correction in our statistical analysis and found no statistical significant associations between *EGFR* SNPs and glioma risk. This may be due to the relatively small sample size, the selection criteria of *EGFR* SNPs (MAF >5%), and the weakness of Bonferroni correction itself. Adjustments for multiple tests, such as Bonferroni correction analysis, are required for medical association studies, but also create more problems than they solve [14]. The main weakness of Bonferroni correction is that the interpretation of a finding depends on the number of other tests performed. True important differences may be deemed non-significant since the likelihood of type II errors are also increased [14]. However, Bonferroni corrections are considered acceptable when performing associations without pre-established hypotheses [14]. Another potential concern was population admixture, which is a known confounding factor for association analysis and can caused inflated type-I errors (false positive). In this study, glioma patients and controls were used in the same hospital to avoid selection bias. However, this bias was unlikely to be of significance because the patient groups did not differ in the distributions of demographic variables and genotype frequencies. We limited all subjects' ethnicity to Han Chinese, and a living area to Xi'an City and its surrounding area, thus there was no substantial population admixture in our study populations.

Our findings in this study provided new evidence for the association between SNPs and haplotypes of the *EGFR* gene and the risk of glioma. The *EGFR* gene is highly variable, and both *EGFR* gene amplification and mutation have been frequently observed in glioblastoma tumors [15]. *EGFR* signaling is initiated by ligand binding to the extracellular ligand-binding domain, which initiates receptor homo-/hetero-dimerization and auto-phosphorylation by the intracellular kinase domain, resulting in receptor activation. The *EGFR* gene was identified to be instrumental in glioma formation by *EGFR* transgenic rats (or mice) that developed cerebellar glioma [16]–[17]. In a previous study, a polymorphism in the 5′-untranslated region of the epidermal growth factor (*EGF*) gene, a natural ligand of the *EGFR*, was identified to play an important role in the pathogenesis of malignant gliomas [18]. They found that patients with the “GA” or “GG” genotype had higher EGF levels, irrespective of the EGFR status, were more likely to recur after surgery, and had a statistically significant shorter overall progression-free survival than patients with the “AA” genotype. Their findings, combined with our results, indicate that *EGFR* pathways may play a key role in the development of glioma.

The *EGFR* gene has been reported as one of the major genes responsible for malignant progression and phenotype reversion of gliomas, and has been used as one of the most important therapeutic targets. However, the mechanism how germline *EGFR* variants contribute to gliomagenesis remains unclear. Since *EGFR* gene amplifications were observed commonly in glioblastoma multiform, we hypothesized that certain mutations or haplotypes rendered the receptor susceptible to *EGFR* amplification. In future studies, to uncover the role of the *EGFR* gene in gliomagenesis, serum *EGFR* expression levels between different mutations or haplotype groups will be compared. We will also investigate the association between germline *EGFR* variants and somatic *EGFR* mutations, and the relationship between serum *EGFR* expression and somatic *EGFR* expression in the same glioma subjects.

In conclusion, our comprehensive analysis of SNPs in the *EGFR* gene suggests that *EGFR* genotypes and haplotypes are associated with glioma risk. These findings indicate that germ-line genetic variants of the *EGFR* gene play a complex role in the development of glioma, and that interactions of loci in the *EGFR* gene may be more important than a single locus. Our study offers important insights into the etiology of glioma.

## Materials and Methods

### Ethics Statement

The use of human tissue and the protocol in this study were strictly conformed to the principles expressed in the Declaration of Helsinki and were approved by the Ethical Committee of Xijing Hospital for approval of research involving human subjects. Signed informed consent was obtained from each participant.

### Study population

In our study population, all analyses were restricted to Han Chinese. A total of 301 patients with glioma between November 2008 and December 2010 were recruited into an ongoing molecular epidemiological study at the Department of Neurosurgery of the Xijing Hospital affiliated with The Fourth Military Medical University (FMMU) in Xi'an city, China. All glioma cases had no previous history of other cancers, or prior chemotherapy or radiotherapy. There were no age, sex, or disease stage restrictions for case recruitment. All patients were recently diagnosed and histologically confirmed to have glioma.

A random sample of 500 healthy unrelated individuals were recruited between June 2010 and August 2010 from the medical examination center at Xijing Hospital, for genetic association research of human complex diseases, such as lung cancer, stomach cancer, and glioma. All of the chosen subjects were Han Chinese living in Xi'an city and its surrounding areas. A detailed recruitment and exclusion criteria were used. Generally, subjects with chronic diseases and conditions involving vital organs (heart, lung, liver, kidney, and brain) and severe endocrinological, metabolic, and nutritional diseases were excluded from this study. The purpose of the above exclusion procedures was to minimize the known environmental and therapeutic factors that influence the variation of human complex diseases. A total of 302 unrelated healthy subjects were recruited as controls in this study.

### Demographic and clinical data

Demographic and personal data were collected through an in-person interview using a standardized epidemiological questionnaire, including age, sex, ethnicity, residential region, smoking status, alcohol use, education status, and family history of cancer. For patients, detailed clinical information was collected through a medical chart review or consultation with treating physicians. Plasma carcinoembryonic antigen and alpha-fetoprotein were tested in control subjects to make sure they did not have any cancers.

### SNP selection and genotyping

Candidate tSNPs in the *EGFR* gene were selected from previously published polymorphisms associated with glioma [12]. Validated tSNPs were selected with a MAF >5% in the HapMap Asian population. A total of 9 tSNPs in the *EGFR* gene were selected for further genotyping. Genomic DNA was extracted from whole blood using the phenol-chloroform extraction method [19]. DNA concentration was measured by spectrometry (DU530 UV/VIS spectrophotometer, Beckman Instruments, Fullerton, CA, USA). A multiplexed SNP MassEXTEND assay was designed with the Sequenom MassARRAY Assay Design 3.0 Software [20]. SNP genotyping was performed using the Sequenom MassARRAY RS1000 with a standard protocol recommended by the manufacturer [20]. Data management and analyses were performed using the Sequenom Typer 4.0 software as previously described [20]–[21].

### Statistical analysis

Statistical analyses were performed using Microsoft Excel and SPSS 16.0 statistical packages (SPSS, Chicago, IL). All *p* values in this study were two-sided. A *p*≤0.05 was considered the threshold for statistical significance. Genotypic frequencies in control subjects for each SNP were tested for departure from HWE using an exact test. Allele frequencies and genotype frequencies for each SNP of glioma patients and control subjects were compared using the χ^2^ test [19], [22]. ORs and 95% CIs were calculated by unconditional logistic regression analyses adjusted for age and sex [23]. We did not divide subjects into subgroups because of the limited sample size. The possibility of sex differences as a source of population sub-structure was evaluated by a genotype test for each SNP in male and female controls, and the number of significant results at the 5% level was compared with the number expected by the χ^2^ test. We did not detect population stratification because all participants' ethnicity was Han Chinese.

The three genetic models (dominant, recessive and additive) were applied by PLINK software (http://pngu.mgh.harvard.edu/purcell/plink/) to assess the association of single tSNPs with the risk of glioma. ORs and 95% CIs were calculated by unconditional logistic regression analyses adjusted for age and sex [23], [24].

We used the Haploview software package (version 4.2) and SHEsis software platform (http://www.nhgg.org/analysis/) for analyses of linkage disequilibrium, haplotype construction, and genetic association at polymorphism loci [25], [26].

## Supporting Information

Table S1 **Raw genotype data of 301 glioma cases.**
(XLS)

Table S2 **Raw genotype data of 302 controls.**
(XLS)

Table S3 **Allele frequency differentiation of rs730437 and rs1468727 between diverse groups of cases with varying aggressive grades.**
(DOC)
