# Supplementary material for: Genetic Variation in the EGFR Gene and the Risk of Glioma in a Chinese Han Population
Source: PLoS One. 2012 May 25;7(5):e37531. doi: 10.1371/journal.pone.0037531 (PMC3360779; doi:10.1371/journal.pone.0037531)
Supplement: Table S3 — Allele frequency differentiation of rs730437 and rs1468727 between diverse groups of cases with varying aggressive grades. (DOC) [file pone.0037531.s003.doc]

Table S3. Allele frequency differentiation of rs730437 and rs1468727 between diverse groups of cases with varying aggressive grades.

|  | rs730437 | | *P* value | rs1468727 | | *P* value |
| --- | --- | --- | --- | --- | --- | --- |
|  | C | A |  | T | C |  |
| astrocytoma (n = 173) | 0.456 | 0.544 | > 0.05 | 0.477 | 0.523 | > 0.05 |
| Ependymoma (n = 20) | 0.400 | 0.600 |  | 0.500 | 0.500 |  |
| Glioblastoma (n = 42) | 0.381 | 0.619 |  | 0.524 | 0.476 |  |
| Oligodendroglioma (n = 9) | 0.389 | 0.611 |  | 0.556 | 0.444 |  |
| Others (n = 57) | 0.439 | 0.561 |  | 0.404 | 0.596 |  |
| Overall (n = 301) | 0.434 | 0.566 |  | 0.504 | 0.496 |  |
